# Central and Peripheral Fatigue in Physical Exercise Explained: A Narrative Review

**DOI:** 10.3390/ijerph19073909

**Published:** 2022-03-25

**Authors:** José Francisco Tornero-Aguilera, Jorge Jimenez-Morcillo, Alejandro Rubio-Zarapuz, Vicente J. Clemente-Suárez

**Affiliations:** 1 Faculty of Sports Sciences, Universidad Europea de Madrid, Tajo Street, s/n, 28670 Madrid, Spain; josefrancisco.tornero@universidadeuropea.es (J.F.T.-A.); jorge.jimenez2@universidadeuropea.es (J.J.-M.); alekeka8j99@hotmail.com (A.R.-Z.); 2 Studies Centre in Applied Combat (CESCA), 45007 Toledo, Spain; 3 Grupo de Investigación en Cultura, Educación y Sociedad, Universidad de la Costa, Barranquilla 080002, Colombia

**Keywords:** psychophysiology, muscle fatigue, muscle activation, cognition, gender

## Abstract

The study of the origin and implications of fatigue in exercise has been widely investigated, but not completely understood given the complex multifactorial mechanisms involved. Then, it is essential to understand the fatigue mechanism to help trainers and physicians to prescribe an adequate training load. The present narrative review aims to analyze the multifactorial factors of fatigue in physical exercise. To reach this aim, a consensus and critical review were performed using both primary sources, such as scientific articles, and secondary ones, such as bibliographic indexes, web pages, and databases. The main search engines were PubMed, SciELO, and Google Scholar. Central and peripheral fatigue are two unison constructs part of the Integrative Governor theory, in which both psychological and physiological drives and requirements are underpinned by homeostatic principles. The relative activity of each one is regulated by dynamic negative feedback activity, as the fundamental general operational controller. Fatigue is conditioned by factors such as gender, affecting men and women differently. Sleep deprivation or psychological disturbances caused, for example, by stress, can affect neural activation patterns, realigning them and slowing down simple mental operations in the context of fatigue. Then, fatigue can have different origins not only related with physiological factors. Therefore, all these prisms must be considered for future approaches from sport and clinical perspectives.

## 1. Introduction

Fatigue is defined as an internal homeostasis breakdown caused by an increase in energy production demanded by an external stimulus. Fatigue can be generally defined as a decrease in physical performance related to a rise within the real/perceived difficulty of a task or exercise, as well as the inability of the muscles to keep up with the specified level of strength during exercises [1]. In sports sciences, the stimulus would be the physical activity, leading to the accumulation of certain metabolites within the muscle fibers or an inadequate motor command in the motor cortex [2].

Fatigue occurs due to the impairment of one or several physiological processes that allow muscle fibers to generate force. This process is known as the “task-dependent factors”, and is one of the principles that have emerged in this era [1]. This term refers to there being no single justifying cause of the etiology of fatigue, since it is considered as a gradual process that involves several and complex physiological changes inside and outside the muscle, which occur before and during mechanical failure [3]. McKenna et al. are some of the first authors to describe that the etiology of fatigue comes from two main roads, either the central nervous system (CNS) by means of central fatigue or the peripheral nervous system, which involves the muscles; thus, peripheral fatigue [4]. In this line, CNS fatigue can be defined as a decrease in the voluntary activation of muscles, directly related to a decrease in the frequency and synchronization of motoneurons, and a reduced drive from the motor cortex. Peripheral fatigue is the decrease in the contractile strength of muscle fibers with changes in the mechanisms underlying the transmission of muscle action potentials [5].

However, fatigue has been considered as a subjective experience in which subjective perception is in turn influenced by a great variety of aspects. Gender is one of them, given the morphological and structural differences between both sexes, in which women have a higher efficiency in lipid oxidation but a lower efficiency in the anaerobic lactic pathways; therefore, less efficient in rapid tests than men. The interpretation of effort and stress differs when faced with the same load, the perception being greater on the part of women. Likewise, the perception of fatigue is greater in women [6,7]. Finally, one of the diseases most closely related to fatigue, fibromyalgia, affects a significantly higher proportion of women than men. Therefore, in relation to fatigue, its etiology, perception, and differences between sexes are obvious [6,7,8]. The type and modality of sports are elements that affect the etiology of fatigue. For example, ultra-endurance events have a significant component of central nervous system fatigue compared to shorter athletic activities or events [9], and explosive or team sports show a wide range of both peripheral and central pathway factors [10,11,12]. Personal problems, anxiety, a reduction in arousal activity, or less vigorous capacity are associated with different brain activation patterns and CNS fatigue [13,14,15]. External factors or elements related to the somatization of stress or a stressful stimulus, such as sleep deprivation, lead to alterations in perception, impairing the subjective perception of fatigue and increasing CNS fatigue [16]. Furthermore, in a stressful or extreme situation, before a stressor stimulus of such magnitude in which danger to physical integrity or life is perceived, the fight–flight mechanism is activated. This leads to alterations in effort and fatigue perception, meaning that despite the high levels of metabolites such as blood lactate, over the anaerobic threshold, the perception of effort is relative to an effort of medium–low intensity [17]. Thus, central or peripheral fatigue, its presence, appearance, gradient, and origin depends on gender, sport type, and psychometric, emotional, psychological, and contextual factors. Therefore, for a better understanding of this complex phenomenon, we conducted the present research to analyze the multifactorial mechanisms and factors that affect the presence and appearance of fatigue.

## 2. Materials and Methods

The protocol was based on a literature search using primary sources, such as scientific articles, and secondary ones, such as bibliographic indexes and databases. Thus, we used PubMed, Scopus, Embase, Science Direct, Sports Discuss, ResearchGate, and the Web of Science using MeSH-compliant keywords, including fatigue, extenuation, physical fatigue, psychological fatigue, sport, endurance sports, anaerobic sports, team sports, gender, central fatigue, peripheral fatigue, psychophysiological, and response. Articles published from 1 January 2000 to 1 December 2021 were included. For inclusion criteria, authors screened the titles and abstracts of all retrieved manuscripts; then, exclusion criteria were applied if: (i) studies used old data (out of the proposed timeline); (ii) had inappropriate topics and were not pertinent to the focused purpose of the study; (iii) were not in English. Extraction of information was performed by the same authors who conducted the study selection. Then, studies were selected independently and the results were discussed to create the present narrative review.

## 3. Etiology of Central Nervous System and Peripheral Fatigue

### 3.1. Central Nervous System Fatigue

Central fatigue is defined as a deficient drive of motor cortical output attenuating performance or even stopping the activity, whereby inhibitory and excitatory processes are affected [18]. Traditionally, authors thought that the origin of central fatigue was caused at any point of the neuromuscular transmission that affects cross bridges; however, recent studies suggest that it is in the brain where central fatigue originates [5]. Being one of the most important elements, biochemical changes produced include the accumulation of extracellular serotonin during exercise practice [2], or other molecules such as gamma-aminobutyric acid, glutamate, or dopamine.

Another factor that explains the appearance of central fatigue is the changes that occur at the spinal cord level regarding the afferent input of the neuromuscular spindles and tendon organs, including Golgi structures and nerve fiber circuits III and IV [19]. There are also areas of the brain that, through a decrease in vascular resistance and an increase in perfusion, manage to maintain homeostasis, including the vestibular nuclear area, cerebellar ventral vermis, and floccular lobe, cardiorespiratory control (medulla and pons), and vision (dorsal occipital cortex, superior colliculi, and lateral geniculate body). Meanwhile, other regions have not been recorded to alter their function during exercise. Regions related to hearing (cochlear nuclei, inferior colliculi, and temporal cortex) and smell (olfactory bulbs and rhinencephalon) are unaltered by exercise. However, the blood flow to the brain is increased in areas related to the integration of sensory input from skeletal muscle. These regions are the anterior and dorsal cerebellar vermis and the maintenance of equilibrium (vestibular nuclei) [20]. Another important brain structure involved in central nervous system (CNS) fatigue is the insula. The right part is associated with the sympathetic activity of the central nervous system and the left with the parasympathetic. In addition, the activity of the insular cortex is associated with the level of volitional exertion related to exercise [21,22].

In addition to impairing physical performance, CNS fatigue also causes cognitive fatigue phenomena, which is associated with behavioral and mood disorders. Prolonging an acute state of CNS fatigue can lead to sleep disturbances, depression, pain, the feeling of fatigue, difficulty in maintaining cognitive vigilance, and problems maintaining mental attention [23]. Evidence from neuroimaging studies supports a model whereby CNS fatigue was described as the failure of the nonmotor function of a network comprising the basal ganglia and its regions (e.g., the dorsolateral prefrontal cortex and thalamus), hypotheses that would explain the psychological changes associated with CNS fatigue [24]. Thus, given the importance of CNS fatigue, field tools have recently begun to be implemented. One of the methods to measure and quantify CNS fatigue and cognitive function is the critical flicker fusion threshold (CFFT). In 1952, Simonson and Brožec already showed the relationship that existed among the CFFT and the level of activation cortical and CNS fatigue, postulating that a decrease in the CFFT would be related to an increase in CNS fatigue; however, recent investigations suggest that the CFFT would only be valid in measuring the level of activation cortical. The valuation of the CFFT has been used for the assessment of fatigue of the CNS by various authors, considering the CFFT within the field of sports and psychology, a system valid for this task [25,26,27]. Coaches could modify the training protocol according to the individual cortical arousal response to improve training efficiency and prevent injuries.

However, it is the alterations in brain biochemistry that derive and explain the etiology of CNS fatigue. Alterations in the concentrations of serotonin, dopamine, glutamate, and gamma-aminobutyric acid are discussed below.

#### 3.1.1. Serotonin

Anatomically, serotonin’s production originates at the level of the rostral raphe nuclei, which is connected to the dendrites and somas of spinal motor neurons by means of monosynaptic neurons. Then, its release takes place in the dorsolateral funiculus. Its implication in the fatigue process is related to its accumulation at the extracellular level due to dopaminergic activity, setting the serotonin/dopamine ratio. In addition, serotonergic projections innervate areas of the hypothalamus and the thermoregulatory center; thus, a change in the activity of these neurons may be expected to contribute to the control of body temperature both at rest and during exercise. It has been reported that during the increase in exercise intensity, serotonergic activity increases, producing a feeling of lethargy, a loss of the neural drive, and, consequently, loss of motor unit recruitment [2]. However, serotonin is unable to cross the blood–brain barrier during moderate-intensity exercise; therefore, cerebral neurons are required to synthesize it for themselves.

It is necessary for the tryptophan available in the bloodstream to be synthesized by the tryptophan hydroxylase enzyme and converted into serotonin; thus, supplying the serotonergic neurons. Normally, tryptophan travels through the bloodstream bound to the albumin, where there is also a smaller amount of free tryptophan. Along with this small bioavailable amount, lipolysis is induced during physical exercise, consequently releasing free fatty acids from the adipose tissue. Consequently, both free tryptophan and free fatty acids in the bloodstream increase, along with the blood flow to the brain, which facilitates its bioavailability for serotonin synthesis. Thus, serotonin brain levels increase and provoke lethargy and tiredness sensations due to the biochemistry changes in several brain regions [2].

Under conditions of physical effort, the arterial cerebral blood flow (CBF) increases, especially in the internal carotid artery, suggesting an increase in blood flow along with different parts of the brain. This increase in CBF is conditioned by a carbon dioxide blood pressure rise (PaCO_2_). Therefore, the increase in the middle cerebral artery means blood velocity (MCAV) reported associated with physical effort appears to depend on the increase in the cardiac output (CO), as has been demonstrated in subjects who took beta-blockers or in patients suffering from heart disease. Near-infrared spectroscopy-determined cerebral oxygenation supports the alterations in MCAV during exercise. Moreover, the cerebrovascular CO_2_ response is shorter in a standing position than in a sitting position. This fact could explain the progressive reduction in CO. However, the use of the Kety–Schmidt technique shows that during the practice of exercise, the global cerebral blood flow remains constant. One limitation of the Kety–Schmidt method for measuring CBF is that it does not consider either the blood flow of the two internal jugular veins or their drainage. While the regional cerebral uptake of oxygen increases during exercise, systemic oxygen levels are kept constant. Yet, during high-intensity exercise, lactate is taken up by the brain, and oxygen uptake also increases. Moreover, at the beginning of the recovering period, immediately following exercise, brain glucose and oxygen uptake are elevated, and lactate uptake remains high. The maintained substrate uptake by the brain after exercise appears to be related to the key role that plays brain glycogen in cerebral activation. However, the final use of brain substrate has not yet been determined [28].

Furthermore, the release of serotonin is also influenced by other neurotransmitters, such as glutamate, GABA (γ-aminobutyric acid or gamma-aminobutyric acid), dopamine, and the availability of glucose. Additionally, serotonin accumulation has been reported to activate 5HT1A autoreceptors. At clinically relevant doses, selective serotonin (5-HT) reuptake inhibitors (SSRIs) and MAO inhibitors (MAOIs) increase the extracellular concentration of 5-HT in the midbrain raphe nuclei, thereby activating inhibitory somatodendritic 5-HT(1A) autoreceptors. In consequence, the activity of 5-HT receptors is reduced and the increase in the quantity of extracellular 5-HT receptors in the forebrain is decreased. Overriding this feedback by using antagonists of 5-HT(1A) autoreceptors permits SSRIs to produce a marked increase in extracellular 5-HT in the forebrain. Hence, combined treatment with an SSRI and a 5-HT(1A) antagonist increases the extracellular concentration of 5-HT even more than using the drug in isolation. During a prolonged release, a negative influence on the firing motor neuron occurs. The most important fact related to serotonin-induced fatigue is closely related to its receptors. Under normal conditions, the 5HT2 receptors, located in the somatodendritic compartment of neurons, have an excitatory role; however, when they are saturated, serotonin binds to 5HT1a receptors located in the initial segment of the axon and have an inhibitory function. This progression is the key factor in serotonin-induced fatigue [28].

#### 3.1.2. Dopamine

Dopamine (DA; 3,4-dihydroxy-phenylethylamine) is another neurotransmitter involved in the central fatigue mechanisms synthesized from the amino acid tyrosine that crosses the blood–brain barrier, transformed into L-3,4dihydroxyphenylalanine (L-DOPA) by tyrosine hydroxylase, and, then, to DA by dopadecarboxylase. DA is released from the terminal nerve and binds to one of five DA receptors, which are divided into two families: D1-like (containing D1 and D5 receptors) and D2-like (with D2, D3, and D4 receptors). The secretion of dopamine is related to producing a delay to fatigue and exhaustion [29,30]. Dopamine neurotransmission during exercise is a potential mechanism in inducing fatigue. For instance, a reduction in dopamine secretion from the SNpc (substantia nigra pars compacta) can damage the activation of the basal ganglia and reduce the stimulation of the cortex motor, contributing to the onset of central fatigue. It has been reported that 6 weeks of wheel running was sufficient to increase the genetic expression of tyrosine hydroxylase and reduce the expression of D2 autoreceptors in the SNpc. In addition, 6 weeks of wheel running increased D2 postsynaptic autoreceptors in the putamen caudate. However, the incredible amount of dopamine and its release during physical activity are later restricted in areas of the brain rich in dopaminergic innervations [31]. The process of the neurotransmission of dopamine is one of the most relevant in the onset of exercise-induced mental and central fatigue [32].

#### 3.1.3. Glutamate

In relation to CNS fatigue, the most important changes are also associated with the declining velocity of the potential action in the axon and the consequent loss of the activation of the muscle fiber. Additionally, the action potential of cells within the cerebral motor cortex might change during the course of maintained motor tasks [18]. In this line, the extracellular fluid lacks an enzyme to break down glutamate [33]. Therefore, in the CNS, glutamate homeostasis is primarily regulated through sodium-dependent excitatory amino acid transporters (EAATs) [33]. The most important among them is glutamate transporter 1 (GLT1), which us responsible for transporting 95% of glutamate and its mobilization [34].

Furthermore, there is a strong association between the concentration of cerebral ischemia, (understood as a decrease in cerebral blood perfusion) and the accumulation of extracellular glutamate [27]. The GLT-1-mediated uptake of glutamate into astrocytes during the activation of neurons can specifically regulate the production of lactic acid, and glutamate can be used in lactate production [27,35]. It has been reported that in physical exercise performed until exhaustion, GLT 1 levels decrease in the supplementary motor area; consequently, this increases glutamate and decreases lactate, both at the extracellular level. The same authors concluded that injecting selective GLT 1 inhibitor in rats into the ventricular brain led to an increase in the concentration of glutamate, leading rats to fatigue [36]. Glutamate is synthesized from carbon skeletons derived from carbohydrates substrates, as long as an amino group is available [37]. Glutamate has been implicated in exercise-induced fatigue, but the underlying mechanism is still unknown. Animal studies aim to determine whether glutamate transporter-1 (GLT-1) has a key role in the regulation of exercise-induced fatigue. The release of GLT-1 and glial fibrillary acidic protein (GFAP) in the supplementary motor area of rats taken to exhaustion was studied by immunohistochemistry. The results showed that GLT-1 release was dampened in rats subjected to exercise-induced fatigue. The inhibition of GLT-1-supplying dihydrokainate and the transcriptional inhibition of GLT-1-providing antisense oligodeoxynucleotides resulted in a decrease in exercise endurance, followed by an increase in the extracellular glutamate concentration and decrease in the extracellular lactate concentration. Additionally, the release of GLT-1 in the supplementary motor appeared to decrease after a vigorous exercise protocol, followed by an increase in the extracellular glutamate concentration. The downregulation of extracellular lactate may be associated with the onset of fatigue.

#### 3.1.4. Gamma-Aminobutyric Acid

Gamma-aminobutyric acid (GABA) is synthesized from glutamic acid decarboxylase (GAD65 and GAD67) in the central nervous system. GAD67 is primarily responsible for 90% of GABA production. On the other hand, GAD65 activates temporally certain biochemical elements that improve GABA concentrations for a faster modulation.

The inhibitory neurotransmitter γ-aminobutyric acid (GABA) is synthesized by two isoforms of the enzyme glutamic acid decarboxylase (GAD): GAD65 and GAD67. Whereas GAD67 is constitutively active and produces >90% of GABA in the central nervous system, GAD65 is temporarily activated and increases GABA concentration to inhibit neurotransmission. Hydrophobic lipid modifications of the GAD65 protein also produce changes in the Golgi membranes and synaptic vesicles in neuroendocrine cells. In contrast, the GAD67 protein is hydrophilic, and in association with the GAD 65 protein through a heterodimerization process, it maintains the integrity of the membrane. The increase in the secretion of GAD 65 is associated with the practice of physical exercise. This increase in secretion also acts as a sympathetic modulator, regulating central fatigue levels. There is a second mechanism that mediates robust membrane attachment, axonal conduction, and the presynaptic clustering of GAD67, but is independent of GAD65. This pathway is canceled by leucine-103 to proline mutation that modifies the conformation of the N-terminal domain but does not affect the GAD65-dependent membrane attachment of GAD67. Then, two different pathways are formed, i.e., the active part of GAD67 to presynaptic clusters to facilitate the accumulation of GABA for rapid delivery into synapses. While exercising, GABA concentrations after a HIIT session tend to increase exponentially in the cortex sensorimotor. The significant increase is positively correlated with an increase in blood lactate, whereas the correlation with lactate levels may relate to the metabolic fate of exercise-derived lactate that crosses the blood–brain barrier, which then accumulates as brain lactate [38]. It has been reported that after a HIIT session, GABA concentrations increase significantly in the sensorimotor cortex. This fact is related to the increase in blood lactate levels, whereas the correlation with lactate levels may relate to the metabolic fate of exercise-derived lactate that crosses the blood–brain barrier [37,38].

### 3.2. Peripheral Fatigue

Peripheral fatigue is defined as the reduction in the efficacy of the neuromuscular junction and processes beyond the neuromuscular function as metabolic and biochemical changes within the muscle [18,39]. The accumulation of metabolites in the bloodstream derived from reactive oxygen species (ROS), such as inorganic phosphates, calcium ions, lactate, ADP, magnesium, and the depletion of glycogen deposits is a factor that breaks homeostasis [40]. This type of fatigue is especially acute in high-intensity exercise, where the accumulation of metabolites could alter the interaction of the actin and myosin cross-bridges. As a direct result, the activity of the ATPase enzyme is reduced, keeping a proportional relationship with the rate of contraction. The metabolic environment in which fatigue develops, for both peripheral and central fatigue, is acidosis with low pH levels, where there is an accumulation of phosphates. Both have a synergistic role in reducing the ability to generate force from the muscle fiber [41].

Peripheral fatigue has a strong correlation with changes in the internal environment (homeostasis). The most important changes during exercise occur when the onset blood lactate accumulation (OBLA) is exceeded, including the accumulation of lactate, hydrogen, and ammonia in the bloodstream. Additionally, the accumulation of heat can lead to a state of dehydration. The accumulation of phosphates and hydrogens in the sarcoplasm can reduce the force of contraction due to the inhibition of the interaction of the cross-bridges. Calcium reuptake from the sarcoplasmic reticulum may also be affected in relation to energetic stores, a decline in glycogen stores, and (in extreme cases) a decline in blood glucose levels. Even a short-lasting decline in blood glucose might seriously interfere with CNS functions. Furthermore, fatigue-induced exercise can also affect the speed of the action potential throughout the sarcolemma, probably due to the biochemical changes produced in the environment of the muscle fiber. Another important factor is the efflux incensement of potassium ions (K+) from muscle fibers. The increase in potassium in the lumen of the T-tubules may lead to a block of the tubular action potential and, hence, less force due to a depression in excitation–contraction coupling [18].

The same occurs with the accumulation of extracellular potassium due to disturbances in the cell’s sodium–potassium pump induced by fatigue [42]. Furthermore, it has been shown that the replacement of electrolytes is faster than the recovery of PH during fatigue situations, also affecting muscle contractions in a negative form [42,43,44,45].

There is also evidence of an inflammatory response to exercise and peripheral fatigue. The underlying mechanisms of fatigue appear to rely on the neuroinflammatory pathways [46]. The local response of muscle production of IL6 during exercise is associated with the regulation in glucose homeostasis as an energy substrate; it is directly proportional to the duration of the exercise [47]. Furthermore, the peak of local muscle IL6 IL1Ra production occurs immediately after exercise, this peak has a positive correlation with the intensity of the exercise. Nevertheless, IL6 production is not associated with muscle damage [48]. Other results are associated with the release of IL6 with muscle damage caused by fatigue from the immediate start of exercise [48].

Continuing on, the mechanism of peripheral fatigue may be reduced to an acute loss of electrical conduction from muscle membrane to tubule system, reducing calcium release from the sarcoplasmic reticulum. Alterations in the cross-bridge cycling with an altered metabolism of actin and myosin a and decreased reuptake of calcium by the sarcoplasmic reticulum lead to bioenergetic failure [49].

#### 3.2.1. What Happens inside the Muscle?

Apart from these physiological changes that occur because of peripheral fatigue, there are metabolic, neuromuscular, and mechanical consequences in muscle cells that originate from fatigue. These are related to three main factors:

(i) Failure in energy metabolism as the myocyte cannot continue resynthesizing ATP.
(ii) Inefficiency in the contraction coupling mechanism due to an impairment in the number or functionality of the actin and myosin cross-bridges.
(iii) Metabolic acidosis produced by the intramuscular accumulation of Pi and hydrogen ions.

The failure in energy metabolism happens when the muscle consumes ATP at a faster rate than it can resynthesize. When there is a decrease in muscle phosphocreatine deposits, there is an increase in muscle creatine and circulating phosphates. In turn, adenosine diphosphate (ADP) levels are increased along with adenosine monophosphate (AMP) concentrations. AMP is metabolized by AMP deaminase, leaving it phosphate-free and able to bind to the ADP molecule to form ATP; thus, supplying muscle tissue [50,51]. The excitation coupling mechanism is affected in different ways. The first one is the damage that is produced, especially in eccentric contractions, in the protein junctophilin involved in the opposition between the T-tubule and the sarcoplasmic reticulum [4]. Secondly, in maximum-intensity contractile actions, what is known as the saturation of the ATP buffering systems occurs, causing the accumulation of inorganic phosphates (Pi) and hydrogen ions (H+), affecting the number of cross-bridges and their functionality (affinity of actin for myosin). Due to this and together with the high ratios in the hydrolysis of ATP, a drop in muscle pH is generated, generating a context of acidosis (these drops can leave the pH at approximately 6.2–6.5 during maximum contractions). The Metabolic acidosis is related to the accumulation of Pi and H+, which has a negative effect on the affinity of the thin filaments with calcium, directly reducing its release from the sarcoplasmic reticulum and, therefore, its mycoplasma concentration, with the consequent reduction in the force production [52].

#### 3.2.2. Thus, Who Is the Bad Guy?

**Figure 1:**
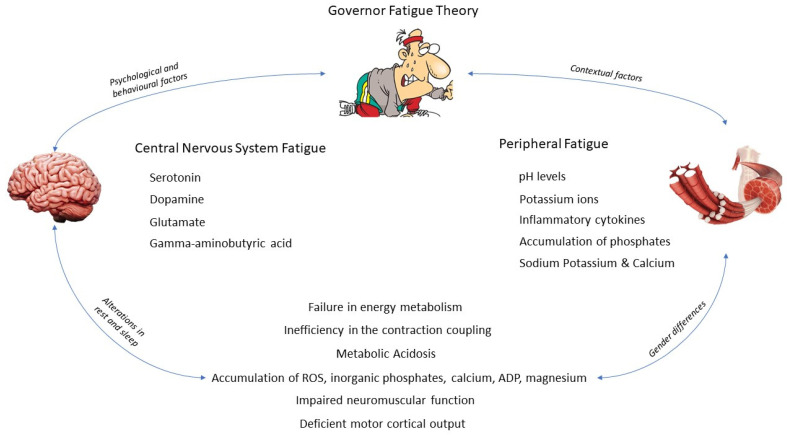
Factors affecting governor fatigue theory.

The fatigue construct cannot be broken down into parts. The current scientific literature suggests that ‘central’ or ‘peripheral’ mechanisms are both theoretical constructs that have ‘straight-jacketed’. The adjustment of both constructs is produced by competition between homeostatic, physiological, and psychological factors. This review intends to develop the Governor theory and relate it to the constructs mentioned above. We suggest that both psychological and physiological drives and requirements are underpinned by homeostatic principles, directed by a negative feedback signal, acting as the general operational controller. The activity in all systems would be based on fluctuations resulting in information, and a comparison of this fluctuant information with either prior information, current activity, or activity templates would create efferent responses that change the activity along with the different systems in a dynamic way. Metabolic or physiological modifications in a single system are always the result of fluctuations taking place outside the system itself. This external activity generates a behavioral history; however, these behaviors are produced by voluntary changes rather than predetermined sensory patterns. Then, fatigue is a complex multifactorial phenomenon in which not only one organic system is involved, but it is a manifestation with implications at different psychological and physiological levels (Figure 1).

## 4. Psychological and Behavioral Modifications and Conditioning Factors

### 4.1. The Fight-or-Flight Response

CNS and peripheral fatigue are not only manifested at a physiological level. There is a close relationship between the information processes of the brain and the behaviors related to fatigue itself. For instance, a previous activation of the sympathetic nervous system from the adrenal medulla elicits a stress response, without the need for the onset of fatigue. This is known as the fight–flight response. The fight-or-flight response is a concept developed by Walter B. Cannon during his studies on the secretion of epinephrine from the adrenal medulla of laboratory animals [53]. This concept was an outgrowth of his studies of homeostatic mechanisms, particularly as they related to the sympathetic–adrenal medulla system. Cannon thought that the sympathetic nervous system and the adrenal medulla operated as a functional unit, with epinephrine as the chemical messenger. He did not understand that the postganglionic sympathetic nerves utilized norepinephrine as a chemical transmitter. Cannon’s research legacy is a rich one, and his work is still cited frequently by contemporary researchers in the field of stress.

The fight-or-flight response itself inhibits fatigue. The mechanism that explains it directly relates to the catecholamine storm produced in the brain, increasing attention, alertness, and psychological and physiological preparedness. This leads to increased performance in cognitive tasks and memory, as well as a decrease in muscular and psychological fatigue [54]. Another mechanism that could explain the reduction in fatigue is associated with the release of catecholamines and their effects. The first consists of an increased cardiac output, increasing blood pressure, vasodilating arteries in skeletal muscle, vasoconstricting arteries in the kidney, gut, and skin, and vasoconstricting veins in general, stimulating the lungs to dilate air passages and initiating hyperventilation [55]. Second, the release of catecholamines increases the energy available to muscles and the brain, avoiding the onset of fatigue. Finally, blood vessels in the skin vasoconstrict to shunt blood, preferentially to internal organs, stimulating sweat production [54]. In general terms, this is how the fight–flight reflex and its consequent reduction in fatigue are associated, mainly starring the release of catecholamines.

### 4.2. Alterations in Rest and Sleep

Sleep deprivation may modify psychological responses related to fatigue. Sleeping 7 to 8 h has been reported to be sufficient to maintain psychological behavior and optimal cognitive performance. Some psychological responses associated with fatigue as a cause of poor sleep are a decreased vigilance, reduced decision-making capacity, slower information processing, the rigidity of thought, difficulty assimilating new information, and greater difficulty in carrying out short tasks. The parasympathetic vagal tone is impaired when there is a deprivation of sleep, as the sympathetic nervous system prevails, causing a state of stress. Brain functions associated with pain inhibition have also been shown to be reduced, causing hyperalgesia [15].

In this line, the use of questionnaires and scales seems similar to an optimal tool to measure and quantify stress, wellness, and recovery. Authors have suggested to use them to detect early signs of tiredness or overtraining in sports programs [56]. For example, the Hooper questionnaire based on self-analysis questionnaires involves well-being ratings relative to sleep, stress, fatigue, and delayed muscle onset soreness [56]. Using the Hooper questionnaire, a significant correlation has been observed among the total training load, perceived sleep, stress, fatigue, and muscle soreness [56].

### 4.3. Gender Differences

In relation to both central and peripheral fatigue, there are differences despite the ambiguity in the scientific literature. One of the main constructs is related to how men and women differ in the way they deal with physical symptoms, emotions, and stress due to a different personality structure [57]. Thus, the subjective perception of fatigue would be greater in females [58]. In addition, females’ biological frameworks, such as the menstrual cycle, pregnancy, childbirth, breastfeeding, birth control medicine, and menopause, are strong conditioning factors of both performance, perception, and the appearance of fatigue [57].

Among the differences, women are less fatigable than men, explained due to neuromuscular, physiological, and anatomical differences [8]. Muscular architecture and the transverse portion of the sarcomere of males is larger than that of females, and some muscles have a greater proportional area, and are metabolically and functionally faster [59,60]. In addition, the sex-specific hormones (androgens and estrogens) cause the male muscles to be stronger and more powerful than female ones; thus, being able to produce, once again, a greater force/energy production [61,62,63,64]. In addition, regarding the contractile properties of muscle, it has been reported that the kinetics of calcium in the sarcoplasmic reticulum were slower when compared with men. Specifically, in young women, calcium kinetics were 24% lower than in men of the same age during repeated sprints [65]. As a result, the sex differences and the activity of the calcium ATPase of the sarcoplasmic reticulum support that women have a musculoskeletal system more resistant to fatigue [66].

Differences in the nervous systems between men and women are related to brain physiology, anatomy, and functional activation through the lifespan. These differences include descending inputs from cortical centers [67,68,69]. Women show greater levels in the activation of ipsilateral and bilateral cortical areas than men, and so a greater blood flow and neural activity. However, men exhibit a greater activation of subcortical areas [70,71]. However, muscle fatigue depends on the specificity and the demand of each task. This specificity may differ between a man and a woman because of the sex-related differences within neuromuscular recruitment. For example, some variables that may differ between men and women in relation to the neuromuscular system are the contraction type, speed, and intensity, involved muscle group, amount of motor units, environmental conditions, and state of arousal [72].

In isometric strength-related tasks, in which the frequency and synchronization of motor neurons are essential factors in the production of force, sex differences have been seen. Women tend to be less fatigable than men at the same relative intensity for several muscle groups, including finger and elbow flexors, adductor pollicis, back extensors, the dorsiflexors of the knee, and extensors and respiratory muscles. These sex differences between muscle groups involve a combination of muscular mechanisms, which include muscular properties, perfusion, and fiber proportions [73,74,75].

In relation to dynamic contractions, women tend to present less fatigue than men. This is explained by the maximum initial torque being higher in men due to their stronger condition [76]. However, the intensity of dynamic contraction can alter the sex difference in fatigability because of the progressive increase in the overall load between the 50% and 90% RM [74]. Another factor involved in this process is the decline in maximal force, due to a reduction in cross-bridge strength, in absolute and relative terms. The same occurs with the speed of contraction linked to the speed of cross-bridge binding and the kinetics of calcium within the fiber [77]. Yet, there are no differences between genders in relation to fatigue and muscle length [78].

Another important difference related to muscle fatigue among genders is blood flow and muscle perfusion, especially in low-intensity contractions [8]. Authors suggest that women present better perfusion than men, but it should be taken into account that differences may appear depending on the specificity of the task and the muscle group involved [75,79,80,81,82,83]. During exercise at the same intensity, blood flow is more restricted in men than women, explained due to males having a higher blood pressure of the arteries [72,84]. In this line, the most important arterial reflex (metaboreflex) occurs when there is an increase in the mean arterial pressure due to the activation of sensory fibers by muscle metabolites. During isometric contractions, the metabolic reflex is greater in men than in women [85]. These receptors are located in greater quantity in type I fibers than in type II, yet women have a higher percentage of type I fibers and a higher amount of beta 2 adrenergic receptors, which cause a better vasodilator response to exercise [86]. Consequently, a better vasodilator response may increase muscle perfusion by accumulating fewer metabolites.

In the use of energy substrates, there are no differences in the oxidative capacity or creatine kinase flux between men and women [87]. However, men show a greater glycolytic mechanism, while females, regarding the greater presence of estrogens, show a greater capacity in the lipid metabolism, including greater mRNA levels of muscle lipoprotein lipase, membrane fatty acid transport protein-1, FAT/CD36 protein levels, and citrate synthase, irrespective of training status and age [88,89]. Furthermore, females present a lower accumulation of lactate and its breakdown products, such as inosine monophosphate [90]. These gender differences in musculoskeletal properties can contribute to less fatigue and a faster recovery in women than in men, especially in power and strength exercises [91].

Furthermore, the menstrual cycle is yet another important factor to consider. Women show increased fatigue in the middle of the menstrual cycle when estrogen levels peak [92]. This phase is called the follicular phase, where the levels of sympathetic activity of the central nervous system are higher, coinciding with the highest concentration in estrogen levels during the menstrual cycle. On the other hand, when progesterone levels are higher, there is no noticeable difference in metabolite accumulation, so higher levels of fatigue are not experienced. This phase is labeled as the luteal phase [93]. However, it has been reported in other studies that the time to exhaustion was reduced and the heart rate, ventilation, and perceived exertion were higher in the luteal phase compared to the follicular phase [94].

## 5. What Happens during Exercise?

There are several physiological variables associated with performance (heart rate, blood lactate, maximal strength, oxygen uptake, cardiac output, and aerobic capacity) that may be strongly affected by several factors. One of them is the perception of effort while exercising. In this line, mental fatigue is presented as a performance key. Defined as a decline in the time to exhaustion, an increase in the self-reported power output and an increase in the time in task duration are associated with a higher perception of exertion than in a non-fatigue state [9].

The mechanisms that explain these processes seem to be associated with the noradrenergic neurotransmitter system. Catecholamine secretion increases central fatigue, coinciding with an accelerated increase in perceived exertion. Additionally, several neurotransmitter systems might be implicated in the most important role for dopamine and adenosine; in multiple brain regions such as the prefrontal cortex and the anterior cingulate cortex, the summation of these alterations might explain the impairment in performance in a mentally fatigued state [32]. From a biochemical point of view, there exist several factors which could lead to fatigue during exercise:

(i) Reactive oxygen species produced during a prolonged eccentric activity produce anions, which damage phospholipids in the muscle cell membrane.
(ii) Hydroxyl radicals generated during muscle stress processes also damage other biomolecules, DNA, and lipids [95].
(iii) A low bioavailability of muscle glycogen and the involvement of glycolysis in ATP hydrolysis for energy during long-lasting physical activity activate nucleotide purine metabolism, leading to an accumulation of inosine monophosphate [96].
(iv) Once glycogen is depleted, brain chain amino acids (BCAA) are oxidized to be used in ATP resynthesis; BCAAs follow the same mechanism as free fatty acids to overcome the brain barrier, where they compete with free tryptophan in the bloodstream. When the BCAA/free tryptophan ratio decreases, serotonin may be accumulated in the brain [97,98], producing a feeling of lethargy and a loss of the neural drive [2]; thus, CNS fatigue.

Other authors have suggested that mental fatigue affects performance through the accumulation of adenosine in different brain regions [99]. In this line, adenosine acts in two ways, i.e., by increasing the perception of effort and by impairing motivation or the willingness to maintain effort, likely via interaction with dopamine in the previous cortex cingulate [100]. It is interesting to notice that the role of adenosine associated with fatigue is supported by the ergogenic effect of the potent adenosine antagonist caffeine on cognitive and sports performance. Adenosine acts as a neuromodulator, which inhibits neural activity via the inhibition of the presynaptic release of neurotransmitters, such as dopamine. The part of the brain most affected by the accumulation of adenosine is the cingulate cortex [100].

There is a theoretical model of exercise-induced mental/central fatigue based on interoception and motivation. The cortex prefrontal dorsolateral region causes a prediction which is forwarded to the insula. During exercise, the insula receives that sensory information from Lamine I spinothalamic and nucleus tractus solitarii medullothalamic pathways [101]. This feedback is compared to the prediction previously generated to build up a current awareness state, which is forwarded to the previous cortex cingulate and the ventromedial and lateral regions of the prefrontal cortex [9]. To finish the process, the lateral region of the prefrontal cortex integrates the information and forces you to decide whether or not to stop [9]. This final decision depends on the interaction of black substantia, locus coeruleus, and adrenal systems, responsible for releasing catecholamines, epinephrine, and norepinephrine [95]. The integration of sensory information and its comparison must occur so that fatigue does not appear [102].

Furthermore, it has been reported that due to increased peripheral fatigue, afferent feedback inhibits the central motor drive and limits performance [103]. Among these factors are the fatigue of respiratory muscles, arterial oxyhemoglobin desaturation, and fluctuations in intrathoracic pressure, which impair the cardiac output and promote the sympathetic vasoconstriction in the bloodstream; thus, compromising the O2 transport to working tissues [104]. Other factors that can induce fatigue are determined by hyperthermia, dehydration, and acidosis. Dehydration leads to hyperthermia reducing skin blood flow, sweating rate, and, thus, heat dissipation, limiting the availability of O2 in the exercising muscles [105].

## 6. Conclusions

Central and peripheral fatigue are two unison constructs part of the Integrative Governor theory, in where both psychological and physiological drives and requirements are underpinned by homeostatic principles. The relative activity of each one is regulated by dynamic negative feedback activity as the fundamental general operational controller. Fatigue is conditioned by factors such as gender, affecting men and women differently. Sleep deprivation or psychological disturbances caused, for example, by stress can affect neural activation patterns, realigning them and slowing down simple mental operations in the context of fatigue. Then, fatigue can have different origins not only related to physiological factors. Therefore, all these prisms must be considered for future approaches from sport and clinical perspectives.

## Data Availability

Not applicable.
